# Ovarian hormones modulate multidrug resistance transporters in the ovary

**DOI:** 10.1186/s40834-018-0076-7

**Published:** 2018-11-15

**Authors:** Lynae M Brayboy, Laura O Knapik, Sokunvichet Long, Mollie Westrick, Gary M Wessel

**Affiliations:** 1 Department of Obstetrics and Gynecology then Division of Reproductive Endocrinology and Infertility, Women & Infants Hospital of Rhode Island, 101 Dudley Street, Providence, RI 02905 USA; 2 Alpert Medical School of Brown University, 222 Richmond Street, Providence, RI 02903 USA; 3 Department of Molecular Biology, Cell Biology and Biochemistry, Brown University, 60 Olive Street, Providence, RI 02912 USA; 4 Brown University, 60 Olive Street, Providence, RI 02912 USA; 5 Biological Basis of Behavior Department, University of Pennsylvania, Room 122 425 South University Avenue, Philadelphia, PA 19104 USA

**Keywords:** Estrogen, Ethinyl estradiol, ATP binding cassette (ABC), Multidrug resistance transporter (MDR), P-glycoprotein, Breast Cancer resistance protein (BCRP), Ovary, Oocyte

## Abstract

**Background:**

Multidrug resistance transporters (MDRs) are transmembrane proteins that efflux metabolites and xenobiotics. They are highly conserved in sequence and function in bacteria and eukaryotes and play important roles in cellular homeostasis, as well as in avoidance of antibiotics and cancer therapies. Recent evidence also documents a critical role in reproductive health and in protecting the ovary from environmental toxicant effects. The most well understood MDRs are MDR-1 (P-glycoprotein (P-gp) also known as ABCB1) and BCRP (breast cancer resistance protein) and are both expressed in the ovary. We have previously shown that MDR-1 mRNA steady state expression changes throughout the murine estrous cycle, but expression appears to increase in association with the surge in estradiol during proestrus.

**Methods:**

Here we test the model that MDR-1 and BCRP are regulated by estrogen, the major hormonal product of the ovary. This was performed by administering 6-week-old female mice either sesame oil (vehicle control) or oral ethinyl estradiol at 1 μg, 10 μg, and 100 μg or PROGESTERONE at 0.25mg, 0.5 mg or 1 mg or a combination of both for 5 days. The mice were then sacrificed, and the ovaries were removed and cleaned. Ovaries were used for qPCR, immunoblotting, and immnunolabeling.

**Results:**

We found that oral ethinyl estradiol did not influence the steady state mRNA of MDR-1 or BCRP. Remarkably, the effect on mRNA levels neither increased or decreased in abundance upon estrogen exposures. Conversely, we observed less MDR-1 protein expression in the groups treated with 1 μg and 10 μg, but not 100 μg of ethinyl estradiol compared to controls. MDR-1 and BCRP are both expressed in pre-ovulatory follicles. When we tested progesterone, we found that MDR-1 mRNA increased at the dosages of 0.25 mg and 0.5 mg, but protein expression levels were not statistically significant. Combined oral ethinyl estradiol and progesterone significantly lowered both MDR-1 mRNA and protein.

**Conclusions:**

Progesterone appears to influence MDR-1 transcript levels, or steady state levels. This could have implications for better understanding how MDR-1 can be modulated during times of toxic exposure. Understanding the normal physiology of MDR-1 in the ovary will expand the current knowledge in cancer biology and reproduction.

**Electronic supplementary material:**

The online version of this article (10.1186/s40834-018-0076-7) contains supplementary material, which is available to authorized users.

## Background

### What are MDRs?

Multidrug resistance transporters (MDRs) play vital roles in normal physiology of an organism and are notoriously responsible for evasion of antibiotics in bacteria and in pathologic chemotherapeutic evasion in neoplasms. MDRs are part of the highly conserved transmembrane ATP-binding cassette (ABC) superfamily of proteins and are detected in nearly every organism ranging from prokaryotes to mammals [1]. MDRs are known to function in the effluxing of various endogenous and xenobiotic substrates such as steroids, peptides, polysaccharides, amino acids, phospholipids, bile acids, ions, and drugs. By using adenosine triphosphate (ATP) hydrolysis [2], these transporters can move substrates against concentration gradients [3], making them powerful regulators of cells. Forty-eight genes encode the ABC family in humans and they are subdivided into seven subfamilies (A-G) based on extent of sequence similarities [4]. MDRs are widely studied in the context of tumor and cancer cell lines, as their overexpression in these cells is a selective agent associated with efflux of chemotherapeutic drugs out of cells and decreased efficacy or even failure of chemotherapeutic treatments [5, 6]. The major ABC transporters responsible for resistance to anti-cancer chemotherapeutics include MDR-1 (P-glycoprotein) encoded by the human ABCB1 gene located on chromosome 7q21 [4, 5, 7] and Breast Cancer Resistance Protein (BCRP) encoded by the human ABCG2 gene located on chromosome 4q22 [1, 3, 8–10].

MDR-1 was first identified in rodent cells that displayed reduced sensitivity to chemotherapeutic drugs in 1976 [11] and is a 170-kDa [7] ABC transmembrane transporter capable of effluxing a variety of cationic amphipathic molecules between 100 and 4,000 Da in size [12]. MDR-1 is composed of two bundles of six transmembrane helices that are connected to two ATP-binding domains [4, 5, 7]. Soon after its discovery, MDR-1 was shown to be expressed not only in many different types of cancer but also in many, and diverse, normal tissue [13, 14]. Originally thought to only confer resistance to common chemotherapeutic drugs, such as vinblastine, doxorubicin, and paclitaxel, researchers later discovered that over 300 compounds serve as substrates of MDR-1 alone [15, 16], and have the capacity to interact with many new generations of anti-cancer compounds such as kinase inhibitors [17, 18]. The MDR family is now known to efflux more than 4000 diverse substrates [7]. The mouse has two closely related MDR-1 isoforms: *mdr1a*, which is highly expressed in the blood-brain barrier, liver, kidney, placenta, and intestine, and *mdr1b*, which is highly expressed in the endometrium of pregnant uterus, placenta, liver, adrenal gland, and kidney [19].

Like MDR-1, BCRP is also responsible for causing resistance to many anticancer drugs, but unlike MDR-1, BCRP is hemi-transporter with a domain structure composed of a single nucleotide binding domain and six putative transmembrane helices [20]. Although MDR-1 and BCRP are widely researched in cancer cell lines and are known for their involvement in the metabolism, absorption, distribution, excretion and toxicity of exogenous compounds [6], they are rarely studied in vivo in normal tissues.

### MDRs are important in reproduction

Growing evidence suggests that MDRs are important to maintain normal reproductive physiology. A recent review documents that these effluxers function in numerous reproductive processes from steroidogenesis, gametogenesis, gamete protection, embryogenesis, fetal development, and parturition [21]. However, their normal physiological roles in the oocyte and the ovary are unclear.

### MDRs function in the ovary

We have previously learned that MDR-1 and BCRP are present in both the oocyte and the somatic cells of the ovary, and that MDR-1 mRNA steady state expression changes throughout the murine estrous cycle, especially after the surge in 17β estradiol during proestrus [22, 23]. It is intriguing to speculate that these transporters are not only present in the female gonad, but also are regulated by the major sex steroid hormone of the ovary, estrogen. This premise is supported by data that suggested the presence of an estrogen response element (ERE) in the promoter of MDR-1 that binds to the estrogen-responsive transcription factors, the ERα and ERβ receptors [24].

The mouse ovary produces estradiol and progesterone levels during the estrous cycle [25]. This estrogen and progesterone, and their fluctuation, may therefore influence the ovarian expression of MDR-1 and BCRP to promote normal ovarian function. We therefore set out to test the hypothesis that ovarian MDR-1 and BCRP are regulated by ovarian hormone levels in the body by use of various dosages of oral estradiol and progesterone followed by testing MDR-1 and BCRP expression in the murine ovary.

## Methods

### Ethinyl estradiol and progesterone treatment of mice

Female wild type FVB 6-week old mice were purchased from Taconic Biosciences (Hudson, NY). The mice were housed in individual plastic cages with ad libitum water and food (Rodent Diet 5010; LabDiet, Richmond, Indiana) at 20–26 °C with a 12:12 photoperiod. The mice were allowed to recuperate from shipping for 3 days. The mice were habituated with a sesame oil and sugar combination and then treated for 5 days with the sesame oil, sugar and ethinyl estradiol dosages of 0 μg, 1 μg, 10 μg and 100 μg combinations based on dosages published by Strom et al. 2012 [26], progesterone dosages of 0.25 mg, 0.5 mg and 1 mg and combined dosages of 0.5 mg of progesterone with 1 μg and 10 μg of EE. On the sixth day, the animals were sacrificed by CO_2_ asphyxiation followed by cervical dislocation and bilateral ovariectomy. All experimental procedures were approved under the animal protocol by the Brown University Institutional Animal Care and Use Committee (IACUC) Protocol numbers 1407000080 and 1710000312. Each treatment group had *n* = 4 mice.

### ER alpha and beta KO mice

The knockout animals were not treated with hormones. They were sacrificed at 6 weeks old by CO_2_ asphyxiation followed by cervical dislocation and bilateral ovariectomy. All experimental procedures were approved under the animal protocol by the Brown University Institutional Animal Care and Use Committee (IACUC) Protocol numbers 1407000080 and 1710000312. Each KO group had n = 4 mice.

### RNA extraction and quantitative reverse transcriptase-PCR

Total RNA was extracted from whole mouse ovaries (*n* = 3) using TRIzol reagent (ThermoFisher Scientific Waltham, MA) in accordance with manufacturer’s protocol. RNA was quantified by use of a NanoDrop Spectrophotometer (ThermoFisher Scientific) and cDNA was then synthesized from 0.231μg of total RNA using cDNA Synthesis Kit (ThermoFisher Scientific) according to manufacturer’s instructions. RT qPCR was then performed using ThermoFisher Scientific qPCR Synthesis Kit with mRNA primers for *mdr1b* (F: 5’-CACAGCTTGTCCAGCCAAT-3′ and R: 5’-ACTTCTCGAAGATGGGCAA-3′), *bcrp* (F: 5’-CTAGGGGCCGAGGCTT AT AC-3′ and R: 5’-AGTGTTGCTACAGACACCACA-3′), and *ER-β* (F: 5’-AAACAGAGAGA CCCTGAA-3′ and R: 5’-CCTCTTGGCGCTTGGACTA-3′) with *β-actin* (F: 5’-TCTTGGGT ATGGAATCCTGTGG-3′ and R: 5’-CAGCACTGTGTTGGCATAGAGG-3′) transcript as a standard. The gene *mdr1b* was chosen over *mdr1a* in the experiment because it is more highly expressed in the ovary.

### Western blot analysis

Whole ovaries of *n* = 3 were homogenized in a lysis buffer of 150 mM NaCl, 50 mM Tris, 1 mM EDTA, 1% NP-40, 0.5% sodium doxycholate, 0.1% sodium dodecyl sulfate, 1 mM Na_3_VO_4_, and 1 mM NaF supplemented with complete, Mini EDTA-free Protease Inhibitor (Roche Diagnostics GmbH Mannheim, Germany), and incubated on ice for 30 mins [27]. The tissues then were centrifuged at 13,200 x g for 10 min; the supernatant was collected as a lysate and the proteins were separated under standard reducing conditions by gel electrophoresis using Novex 4–12% Tris-Bis gels (ThermoFisher Scientific, Waltham, MA). Proteins were then transferred to an Immobilon-P PVDF blotting membrane (Sigma-Aldrich, St. Louis, MO) at 30 V for 70 min and the membranes were blocked at room temperature on a gentle rocker for 1 h in 2% BSA diluted in 0.1% *V*/V Tween-20 (TBST). Blots were incubated in 1:3000 dilution of P-gp (ab170904) monoclonal with an expected size of 141 kDa predicted to demonstrate a smear between 150 and 300 kDa due to protein glycosylation (Abcam, Cambridge, United Kingdom) of a 1:1000 dilution of alpha-Tubulin (sc-53030) primary antibody (Santa Cruz Biotechnology, Dallas, TX) overnight at 4 °C. Blots were washed 3 times in TBST and incubated at room temperature for 1 h in 1:3000–1:5000 dilution of the corresponding antibody conjugated in HRP. The blots were then washed 3 times in TBST followed by chemiluminescence detection using the Pierce ECL Western Blotting Substrate detection kit in and exposed to film (ThermoFisher Scientific, Waltham, MA).

### MDR localization in ovarian follicles with immunofluorescence

Whole ovaries were fixed in DietricH’s Solution and then embedded in paraffin blocks. Five-micron sections of these tissues then were rehydrated through a xylene, ethanol series and then rinsed with PBS. Heat-induced epitope retrieval was performed using sodium citrate buffer. Endogenous peroxidase activity was inhibited with a 3% aqueous peroxide solution and then blocked with 10% fetal calf serum 0.1% Tween-20 1% BSA in PBS for 1 h. The antibodies used were rat monoclonal ab24115 (Abcam Cambridge, UK) for BCRP localization and for MDR-1 sC1517 goat polyclonal from (Santa Cruz Biotechnology, Dallas, TX).

### Statistical analyses

All statistical analyses were performed using Prism GraphPad (LaJolla, CA) with a non-parametric t-test with a *p*-value of *p* = 0.05 being significant.

## Results

### Real-time quantitative polymerase chain reaction of MDR-1 and BCRP

**Fig. 1:**
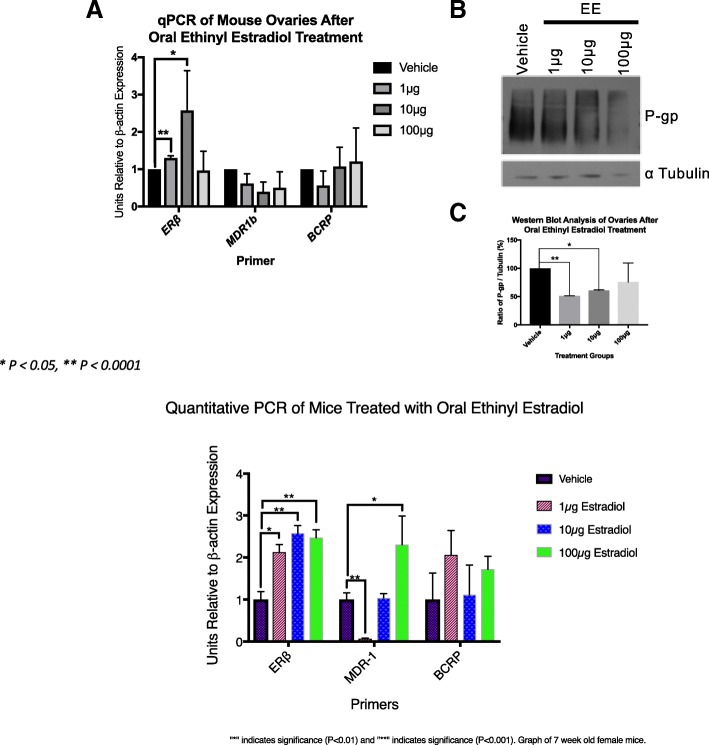
*Estrogen doesn’t modulate steady state mRNA message of MDR-1 but decreases protein level* In this experiment, the control (vehicle only) and estrogen treated animals’ ovaries were removed and placed in TRizol for RNA extraction. RNA was then used to make complementary DNA. Quantitative Real-Time Polymerase Chain Reaction was performed using (ERβ) as a control that indicated the mice did actually receive the appropriate dosing. All samples were standardized to β-actin mRNA. The immunoblots were done by using whole ovary lysates of control and estrogen treated mice. Dosages of 1 μg and 10 μg were significantly decreased compared to controls. BCRP did not show a difference with our qPCR data, but we attempted to do immunoblotting without success due to unsuitable antibodies

**Fig. 2:**
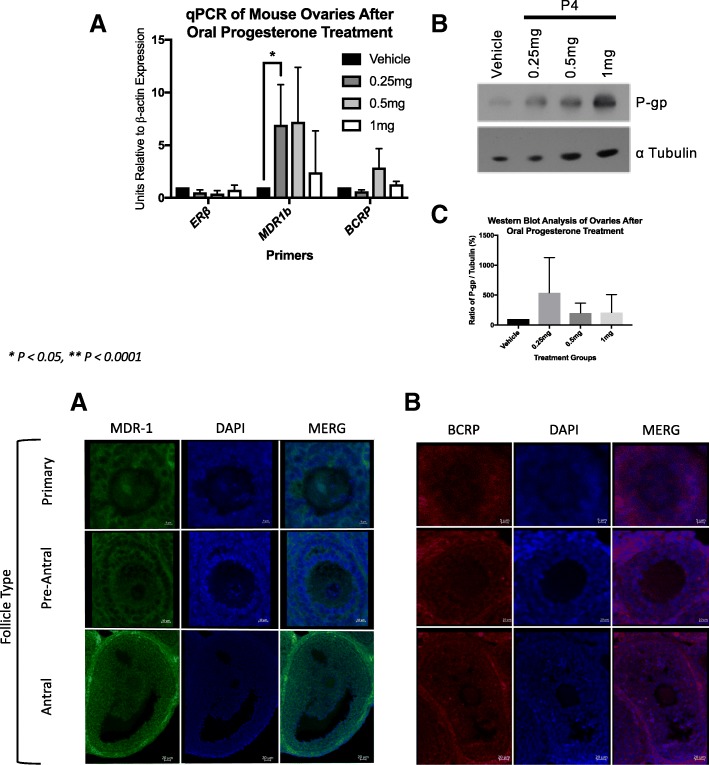
*Progesterone increases steady state mRNA levels of MDR-1 but does not change protein expression* Here in this experiment mice treated with progesterone were sacrificed and the ovaries were used for qPCR as described previously and immunoblot. Significant differences were seen in the treatment groups, 0.25 mg and 0.5 mg, but not 1 mg

**Fig. 3:**
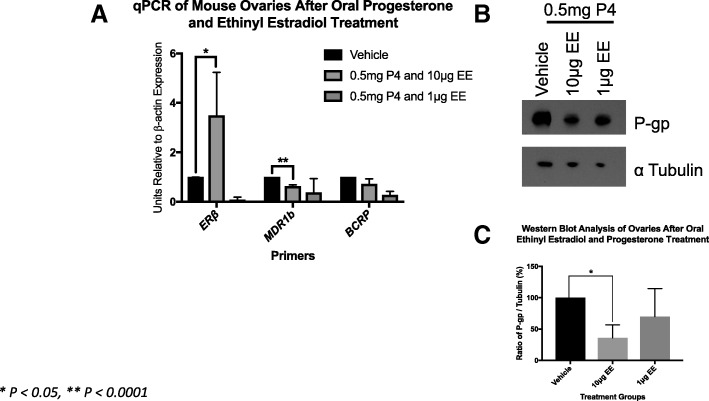
*Progesterone and ethinyl estradiol combined decreases mRNA and protein levels* The qPCR data with 10 μg EE and 0.5 mg of progesterone show that MDR-1 steady state message is significantly decreased as well as protein expression

**Fig. 4:**
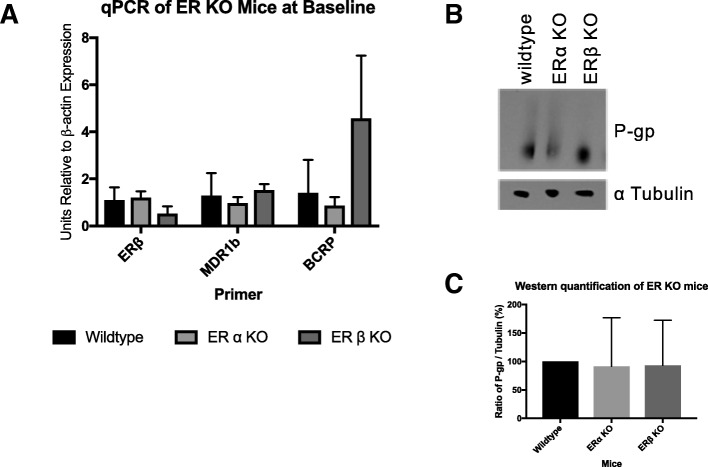
*Loss of the ER alpha or beta receptor does not* MDR-1 expression Both the qPCR and immunoblot data show no significant differences in mRNA and protein expression levels

Ethinyl estradiol either alone or in combination with progesterone did not illicit changes in the steady state levels of MDR-1 or BCRP levels (Fig. 1). Interestingly, progesterone alone at 0.25 mg and 0.5 mg did increase the steady state of MDR-1, but not BCRP (Fig. 2). Combined EE and progesterone decreased the mRNA and protein expression (Fig. 3). Estrogen receptor beta (ERβ) was employed as a positive control, since it is known to be estrogen-responsive. Estrogen Receptor alpha and beta knockout animals did not display changes in the steady state message of MDR-1 or the protein expression (Fig. 4). All samples were standardized to β-actin mRNA.

### Immunoblot of MDR-1 and BCRP

The 1 μg and 10 μg of EE dose seemed to suppress MDR-1 protein expression (Fig. 1). Protein expression with progesterone treatment was unchanged (Fig. 2). The combination of 10 μg EE and 0.5 mg of progesterone also decreased protein expression as did EE alone (Fig. 3). BCRP was technically difficult despite trialing several monoclonal antibodies for BCRP. Shown above is MDR-1 only.

### Immunolocalization of MDR-1 and BCRP

**Fig. 5:**
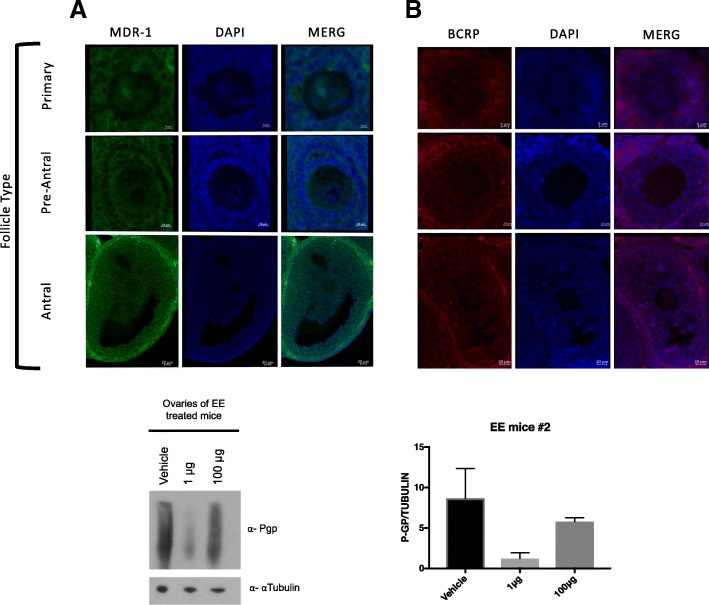
*MDR-1 and BCRP immunolocalization is abundant in preovulatory follicles* Here, immunolocalization was performed on sections of fixed paraffin embedded ovaries of untreated mice. Primary, preantral (secondary), and antral follicles were selected in particular because they reflect growing follicles that are undergoing proliferation and are under the control of Follicles Stimulation Hormone (FSH), which drives estrogen production in the granulosa cells

Here we show that both A) MDR-1 and B) BCRP appear localized to the oocyte, the granulosa cells of primary, preantral follicles, and antral follicles (Fig. 5). MDR-1 localizes more in oocytes compared to BCRP, but both transporters appear to be widely expressed throughout the entire follicle. The follicles are co-labeled with DAPI to display nuclei.

## Discussion

### Estrogen alters MDR-1 in the ovary

The relationship between estrogen and ovarian Multidrug Resistance Transporters (MDRs) could further our understanding of the mechanism by which these transporters function to protect the ovary from xenobiotics and toxicants. Multidrug resistance transporter-1 is abundantly expressed in the oocyte [23] and ovary [22] and responds to endogenous estradiol [22] as well as exogenous estrogen, which we demonstrate here. The steady state message is not increased by high dose ethinyl estradiol, but protein expression was observed to be lower than the animal’s endogenous expression (Fig. 1). This is perhaps explained by the fact that endogenous estrogen may be more efficient at inducing translation as opposed to ethinyl estradiol. How this regulation may work is not known, but protein regulation at the level of translation and turnover is prevalent in the literature, especially for the germ line cells. Another possibility is dosing of estrogen is very important as we see inhibition of steady state message at low doses while increased message at 100 micrograms (Fig. 1). Conversely, exogenous estradiol may have some inhibitory mechanism on MDR-1 translation. However, these data provide further evidence of the importance of estrogen’s regulation of these ovarian transporters. Estrogen’s regulation of *ABCB1* is also supported by our Ingenuity Pathway Analysis modeling data (Additional file 1: Figure S1) which predicts estrogen as an inhibitory regulator. Yet, the drawback of IPA is that this modeling draws only from literature published mostly using cancer cell lines and non-ovarian organs, which may have pathologic or different MDR regulation. Data from Binder et al. 2013 has shown that *Abcb1*, the isoform of MDR-1 in mice that is highly expressed in the ovary, was the gene with the most reduced expression in ERβ knockout mouse granulosa cells versus wild type mice that had been stimulated with PMSG and hCG [28] for superovulation, which normally leads to large surges in estradiol. This granulosa cell expression correlates with our immunofluorescence data (Fig. 5) where we demonstrate that MDR-1 is expressed intensely in the granulosa cells of the follicles, the location of 17β estradiol production. It remains unclear how exactly estrogen interplays with MDR-1 and further experimentation needs to be performed to clarify the exact relationship of estradiol to non-pathologic MDR-1 in the ovary.

We have also previously shown BCRP is expressed in the ovary [22] and while it is reported to have an estrogen response element [29] it does not appear to respond to estradiol fluctuation during the estrous cycle [22] nor to exogenous ethinyl estradiol (Fig. 1). It also appears to be less intensely expressed in pre-ovulatory ooctyes compared to MDR-1 (Fig. 5). These findings taken together reinforce the importance of diverse regulation of the MDR family of genes in reproduction.

### The oral contraceptive pill may modulate MDR activity

Given ethinyl estradiol is not the only hormone in the combined oral contraceptive pill we tested progesterone alone and combined EE and progesterone. Our goal was to investigate how estrogen and progesterone synergistically modulate MDRs thereby demystifying or partly explaining the basic pathophysiology of how the combined oral contraceptive pill protects the ovary from epithelial ovarian carcinogenesis perhaps via Multidrug Resistance Transporters expression and efflux capability. We found that progesterone alone does increase the steady state of mRNA, but does not change protein expression (Fig. 2). However, when estrogen was added in combination of with progesterone the protein levels of MDR-1 were decreased (Fig. 3). This potentially demonstrates estrogen’s dominant effect on MDR-1.

### Other ovarian MDRs need to be evaluated

MDR-1 and BCRP are not the only transporters in the ovarian transcriptome. Data from NCBI shows that there are 23 other transporters in the ovary that may work synergistically or independently with MDR-1 and BCRP. We are currently exploring these other transporters so that we can localize their expression in the ovary through in situ hybridization. Testing the localization and expression of other ovarian MDRs will better help us to understand how the other transporters are regulated in response to all ovarian sex steroid hormones. The eventual goal will be to understand how these transporters may change over the reproductive lifespan as a function of menopause and the lack of estradiol surges.

## Conclusions

MDRs are abundantly expressed in the mammalian ovary and we now show that MDR-1 transcript is regulated by progesterone, but not estrogen. Instead, we found estrogen decreases protein expression alone or in combination with progesterone. Therefore, these transporters can potentially be manipulated to be over-expressed during times of toxic exposure using hormonal medication. The controlled modulation of MDR-1 may have implications for the development of alternative fertility preservation methods. Understanding how these transporters are regulated in normal ovarian physiology has important implications for the field of cancer biology as well as reproduction. Progesterone regulation of MDRs could provide inroads and important information regarding their pathologic transition in cancerous cells.

## Additional file

Additional file 1: *Ingenuity Pathway Analysis Predicts Estrogen Regulation of MDR- 1* Mechanistic network for ESR1 predicted activation Target molecules *ABCB1* (*mdr1a* in Ingenuity Pathway Analysis) and *Abcb1b* were added to the network to depict the predicted relationship between *ESR1*, green color indicates downregulation of these genes. Orange shapes indicate predicted activation, blue shapes indicate predicted inhibition. The prediction relationships which are depicted as lines between molecules; orange color indicates leading to activation, blue color indicates leading to inhibition, yellow color shows findings are inconsistent with the state of downstream molecules and gray colored lines indicate that effect was not predicted [30]. (PDF 308 kb)

## Abbreviations

ABC: ATP Binding Cassette
BCRP: Breast Cancer Resistance Protein
EE: Ethinyl Estradiol
MDR: Multidrug Resistance Transporter
P-gp: P-glycoprotein

## References

[1] Sarkadi B (2006). Human multidrug resistance ABCB and ABCG transporters: participation in a chemoimmunity defense system. Physiol Rev.

[2] Klein I, Sarkadi B, Varadi A (1999). An inventory of the human ABC proteins. Biochim Biophys Acta.

[3] Dean M, Rzhetsky A, Allikmets R (2001). The human ATP-binding cassette (ABC) transporter superfamily. Genome Res.

[4] Szakacs G (2006). Targeting multidrug resistance in cancer. Nat Rev Drug Discov.

[5] Eckford PD, Sharom FJ (2009). ABC efflux pump-based resistance to chemotherapy drugs. Chem Rev.

[6] Leslie EM, Deeley RG, Cole SP (2005). Multidrug resistance proteins: role of P-glycoprotein, MRP1, MRP2, and BCRP (ABCG2) in tissue defense. Toxicol Appl Pharmacol.

[7] Ambudkar SV (1999). Biochemical, cellular, and pharmacological aspects of the multidrug transporter. Annu Rev Pharmacol Toxicol.

[8] Dean M, Annilo T (2005). Evolution of the ATP-binding cassette (ABC) transporter superfamily in vertebrates. Annu Rev Genomics Hum Genet.

[9] Kapse-Mistry S (2014). Nanodrug delivery in reversing multidrug resistance in cancer cells. Front Pharmacol.

[10] Mao Q, Unadkat JD (2015). Role of the breast cancer resistance protein (BCRP/ABCG2) in drug transport--an update. AAPS J.

[11] Juliano RL, Ling V (1976). A surface glycoprotein modulating drug permeability in Chinese hamster ovary cell mutants. Biochim Biophys Acta.

[12] Schinkel AH, Jonker JW (2003). Mammalian drug efflux transporters of the ATP binding cassette (ABC) family: an overview. Adv Drug Deliv Rev.

[13] Kartner N (1985). Detection of P-glycoprotein in multidrug-resistant cell lines by monoclonal antibodies. Nature.

[14] Cordon-Cardo C (1989). Multidrug-resistance gene (P-glycoprotein) is expressed by endothelial cells at blood-brain barrier sites. Proc Natl Acad Sci U S A.

[15] Wang Z (2011). P-glycoprotein substrate models using support vector machines based on a comprehensive data set. J Chem Inf Model.

[16] Chen L (2012). Computational models for predicting substrates or inhibitors of P-glycoprotein. Drug Discov Today.

[17] Hegedus T (2002). Interaction of tyrosine kinase inhibitors with the human multidrug transporter proteins, MDR1 and MRP1. Biochim Biophys Acta.

[18] Wang XK, Fu LW (2010). Interaction of tyrosine kinase inhibitors with the MDR- related ABC transporter proteins. Curr Drug Metab.

[19] Schinkel AH (1997). The physiological function of drug-transporting P-glycoproteins. Semin Cancer Biol.

[20] Doyle LA (1998). A multidrug resistance transporter from human MCF-7 breast cancer cells. Proc Natl Acad Sci U S A.

[21] Bloise E (2016). ATP-binding cassette transporters in reproduction: a new frontier. Hum Reprod Update.

[22] Brayboy LM (2017). Multidrug resistance transporter-1 and breast cancer resistance protein protect against ovarian toxicity, and are essential in ovarian physiology. Reprod Toxicol.

[23] Brayboy LM (2013). Multidrug-resistant transport activity protects oocytes from chemotherapeutic agents and changes during oocyte maturation. Fertil Steril.

[24] Shi JF (2014). ERalpha directly activated the MDR1 transcription to increase paclitaxel-resistance of ERalpha-positive breast cancer cells in vitro and in vivo. Int J Biochem Cell Biol.

[25] McLean AC (2012). Performing vaginal lavage, crystal violet staining, and vaginal cytological evaluation for mouse estrous cycle staging identification. J Vis Exp.

[26] Strom JO (2012). Ovariectomy and 17beta-estradiol replacement in rats and mice: a visual demonstration. J Vis Exp.

[27] Breen SM (2013). Ovulation involves the luteinizing hormone-dependent activation of G(q/11) in granulosa cells. Mol Endocrinol.

[28] Binder AK (2013). The absence of ER-beta results in altered gene expression in ovarian granulosa cells isolated from in vivo preovulatory follicles. Endocrinology.

[29] Ee PL (2004). Identification of a novel estrogen response element in the breast cancer resistance protein (ABCG2) gene. Cancer Res.

[30] Lai AC, Crews CM (2017). Induced protein degradation: an emerging drug discovery paradigm. Nat Rev Drug Discov.

