# Supplementary figures and images for: Ovarian hormones modulate multidrug resistance transporters in the ovary
Source: Contracept Reprod Med. 2018 Nov 15;3:26. doi: 10.1186/s40834-018-0076-7 (PMC6236903; doi:10.1186/s40834-018-0076-7)

# Supplemental Figure 1

ESR1 1

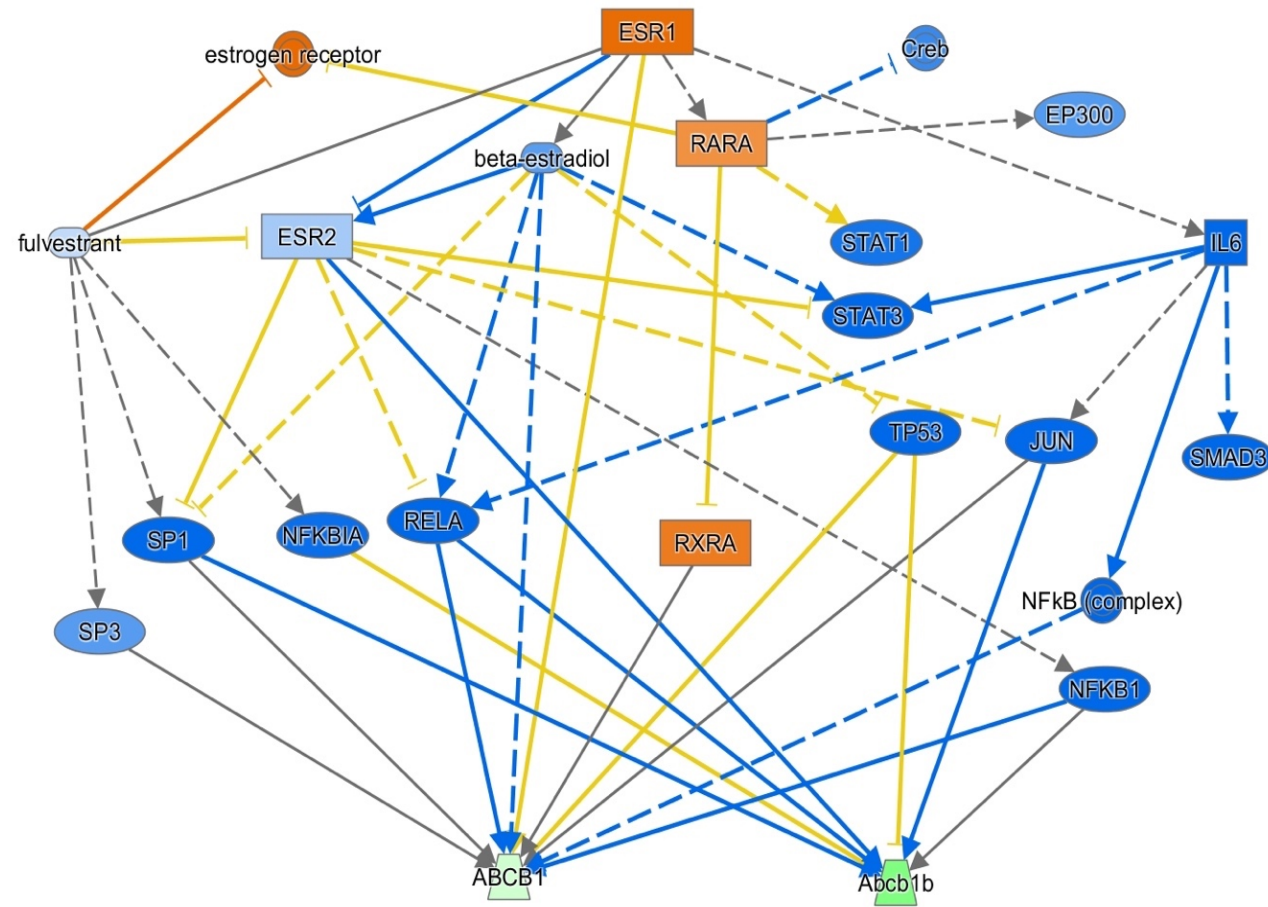

Supplement: Supplementary file 1 — Ingenuity Pathway Analysis Predicts Estrogen Regulation of MDR- 1 Mechanistic network for ESR1 predicted activation Target molecules ABCB1 (mdr1a in Ingenuity Pathway Analysis) and Abcb1b were added to the network to depict the predicted relationship between ESR1, green color indicates downregulation of these genes. Orange shapes indicate predicted activation, blue shapes indicate predicted inhibition. The prediction relationships which are depicted as lines between molecules; orange color indicates leading to activation, blue color indicates leading to inhibition, yellow color shows findings are inconsistent with the state of downstream molecules and gray colored lines indicate that effect was not predicted [30]. (PDF 308 kb) [file 40834_2018_76_MOESM1_ESM.pdf]
